# Improving public awareness and outcomes for oral cancer

**DOI:** 10.4155/fsoa-2016-0004

**Published:** 2016-02-11

**Authors:** Catherine F Poh

**Affiliations:** 1 Faculty of Dentistry, Department of Oral Biological & Medical Sciences, University of British Columbia, Vancouver, BC, Canada

**Keywords:** awareness, oral cancer, oral premalignant lesions, progression, recurrence

## Abstract

**Catherine Poh speaks to G Westcott, Commissioning Editor**: Dr. C Poh gained a dental degree from the National Taiwan University in Taipei, Taiwan and went on to gain her PhD from the University of British Columbia in 1997, looking into molecular biology in oral cancer. In addition to her current role an associate professor in the Faculty of Dentistry at the University of British Columbia, she is a senior clinician scientist at the Integrative Oncology Department of the BC Cancer Agency's Research Centre. She has various other roles, including being active within the Oral Oncology Department at BC Cancer Agency and the Oral Mucosal Disease Program at Vancouver General Hospital. Her research focuses on the application of molecular and imaging tools for screening, detection and management of at-risk oral lesions. She is currently the principal investigator of a Terry Fox Research Institute-funded ($5 million), pan-Canadian multicenter randomized controlled surgical trial.

## Q Can you tell us a little about your career to date, & how you came into oral oncology?

I am a dentist and an oral pathologist by training. I did my PhD training at the University of British Columbia (Canada) and studied molecular biology of oral cancer and premalignant lesions. I had the opportunity to analyze the clinical information from oral cancer patients as well as the pathology and molecular results. With the opportunity to combine knowledge of all three aspects from oral cancer patients, I feel like I am in the right position to answer a lot of questions. I feel that a lot of clinical problems remain to be solved. I was fascinated by the ability to apply basic science to patient care. So I take this as my career goal.

## Q You are often involved with the screening process for oral lesions. What are the current problems faced in this area?

The main problem is that the majority of premalignant lesions and early oral cancer lesions are asymptomatic, so there is no oral pain or discomfort to patients. We know that treatment at an early stage has a better outcome, so screening to identify these at risk lesions early before they become cancer is a major way to improve oral cancer outcome. The problem we face has two aspects: the first is a challenge for clinicians. Oral lesions can be confused with reactive lesions, which are lesions that react to something, for example trauma, infection or an autoimmune condition. In the oral mucosa, we commonly see an autoimmune condition called oral lichen planus, in which the clinical manifestation looks very similar to precancerous or early cancers – even to experienced clinicians. Continuous training on how to identify lesions and distinguish between reactive versus cancerous lesions is very important. In addition, the development of objectives, approaches and tests is critical to improve the efficiency of screening. The other aspect is patient awareness. Many of my patients do not know that the mouth can develop cancer. Patients can therefore be delayed in asking for help, and when they present with cancer, it is often at a late stage. I think that raising public awareness is a matter linked to screening too, because if they are not aware then they will not come forward for screening.

## Q You mention public awareness – how can we use this as a tool to address screening concerns?

We are thinking about this. We want to reach out to the dental community with flyers in the clinics. On a larger scale, we need to raise awareness with bulletin boards or commercial advertisement. Everybody knows a lot about breast cancer, prostate cancer and lung cancer, but many people are not as aware of oral cancer. The prognosis of oral cancer can be really detrimental. One in two patients die within 5 years, so it is at high risk – particularly for those late-stage cancer patients – their survival is as low as 20%. It really is a high fatality and mortality cancer – even for those who survive they can have a lot of functional and esthetical compromise due to the treatment. Awareness really needs to be raised and then people can see this as a disease that they need to be made aware of. Another thing I wanted to mention is that we are trying to raise awareness for young patients. The youngest patients in our center are around 20s. Many epidemiological studies have shown that there is an increasing number of young tongue cancer. This is an awareness that is needed not only to the public but also to our physician colleagues. Many young patients come to the clinic and are treated without knowing that they are also at risk for oral cancer. This can contribute to a time delay for diagnosis. This is a disease that people really need to put attention to!

## Q How close are we to a test for accurately checking risk for low-grade lesions?

In current standards, for a test to be able to reach the public and to be used in the clinical settings, we have to have a retrospective study and then we need to have another cohort to validate the results again. In the test we are developing to triage risk for oral premalignant lesions, we had a chance to conduct a retrospective and a prospective study over 10–15 years of studying. For premalignant lesion studies, it is even harder to accurately test this because these lesions take a long time to evolve into cancer. We published two papers, one in 2002 and one in 2012, and the work from these has transformed into a clinically actionable test that has potential for public use. In 2016 this test will be available. In Canada, however, we have to apply a 3-year business plan to evaluate whether the test is cost-effective before it could be used in our public health system. Another thing we are doing is working with business sectors that allow the test to be available to the public and to benefit patients in need in different jurisdictions.

## Q Can you tell us about your work with the COOLS trial, using blue light to visualize the lesion margins?

This technique is using blue light, not florescence or UV light. We use a filter to filter out all visible light but blue light, which has energy that can pass through the filter. We use this blue light to visualize the tissue: when we shine the light at the tissue, the tissue has gain tissue autofluorescence so we do not need to apply any additional agents to visualize the change. This is a technology that scientists at BC Cancer Agency (BC, Canada) developed. From 2004 we pioneered this test in our oral oncology clinic. We gradually learned that the device can observe the extent of the lesion, which is often not so apparent to our unaided eye. We then started working with our ENT surgeons to visualize tumors in the operating room. We found that the changes seen under the visualization device were not only associated with high-grade pathology but also with high-risk molecular changes. This would have a high risk of coming back if we did not remove it, which can contribute to high local recurrence rate. We now have enough samples and outcomes to observe if the device is really improving local recurrence. In our research, we saw a reduced local recurrence rate from two digits to one. The data were published recently in *JAMA Otolaryngology* [1].

## Q This year you have begun the patient support initiative. Can you tell us a little about that?

We know that not many patients are aware of oral cancer. They also are not sure what they will go through during and after treatment. Treatment often involves surgery and a combined modality, like chemotherapy or radiation. We want to provide a supportive portal, like a website, to provide information for patients and their loved ones. The information will empower them to face the challenges of this disease. We are in the stage of organizing this website, but in the meantime we are trying to fundraise because we need support, particularly in the form of a person that serves as a navigator to guide them through the treatment processes.

## Q What else are you working on at the moment?

The COOLS is a Phase III multicenter randomized control trial funded by the Terry Fox institute. Terry Fox was a young person with cancer of the leg and he ran across Canada from East to West to raise money for cancer research. Unfortunately, he did not finish the run, but it is amazing that it has gained global recognition. It has even become a global event, run not only in Canada, but in Asia. It is amazing how one person can generate sustainable funding to support so much cancer research. This Phase III trial will be completed in May 2016. Hopefully we can validate this result and change current practice – to use this device to control local recurrence of oral cancer. Additionally, during the trial we collected a lot of biosamples and a lot of information for follow-up. We found out that these patients often pass away quickly before they have a chance to develop local recurrence. Many had developed nodal disease, where the cancer had metastasized to their cervical lymph nodes. We have therefore hypothesized that the tumor has two groups: one will not go to lymph node, no matter how large they are and one will. We are looking at how to develop better markers to predict risk those that will metastasize to the node and those that will not. Then we can identify patients and treat them more aggressively before they become late-stage disease.

## Q You work as part of a multi-disciplinary team. How has this led to your success?

All my work is patient oriented: we use clinical problems to drive research hypotheses. I see patients almost every day so I know the urgent need to improve outcomes. In a multidisciplinary team, we can work together with experts in other fields to synthesize thoughts and brainstorm new ideas that solve clinical problems. This can help clinicians, including surgeons, pathologists, dentists, to take better care of their patients and impact patients’ lives. This translational research has a big impact, and brings problems from the clinic to scientists, who have new ideas to improve clinical care.

## Q If you had unlimited resources, what would you do & why?

That would be fantastic! I think I would have an ‘A team’ made up of experts and leaders across different fields. In addition, an important part of conducting research is having sustainable supporting staff. Due to the limitations of funding, if there is a hiccup when we apply for grants, there is a window of time where we sometimes cannot retain our staff. It can sometimes feel like we are training people for other people to use. If there are unlimited resources, we could have sustainable staff to support long-term progress. For our premalignant studies, if we did not have ongoing support from the NIH (MA, USA) we would not have been able to develop this longitudinal trial, observe the patient outcomes and together develop the test. Without over 10 years effort we would not be able to see that work come to real life. It is important for sustainable funding to have sustainable people and in the long run this would really help to tackle this disease.

The opinions expressed in this interview are those of the interviewee and do not necessarily reflect the views of Future Science Ltd.
